# Transfusion transmitted infections – A retrospective analysis from the National Blood Transfusion Service in Eritrea

**DOI:** 10.4314/pamj.v9i1.71219

**Published:** 2011-08-18

**Authors:** Nahom Fessehaye, Durgadas Naik, Tesfay Fessehaye

**Affiliations:** 1 Department of Biomedical and Clinical Sciences, Asmara College of Health Sciences, Asmara, Eritrea

**Keywords:** Enzyme Linked Immunosorbent Assay, Hepatitis B Virus, Hepatitis C Virus, Human Immunodeficiency Virus, Transfusion Transmitted Infection, Blood transfusion, Eritrea

## Abstract

**Background:**

The emergence of transfusion transmitted infection (TTI) especially HIV/AIDS has created a huge obstacle in ensuring blood safety. To assess the situation in Eritrea, we carried out a retrospective study of 29,501 blood donors for the prevalence of TTI's i.e. HIV, HBV, HCV and Syphilis.

**Methods:**

The study population included all donors who donated blood from January 2006 to November 2009. The data was collected from the National Blood Transfusion Services (NTBS) of Eritrea and includes category of donor and result for TTI markers.

**Results:**

A total of 29,501 units of blood were collected from 23,385(79%) voluntary blood donors and the rest 6,116(21%) units were collected from family replacement donors. The over all prevalence of TTI's were 3.8% with 3.5% in voluntary blood donors and 5.1% in family replacement donors. The sero-prevalence for TTI markers were 0.18% HIV, 2.58% HBV, 0.57% HCV and 0.49% Syphilis.

**Conclusion:**

In conclusion, even if the TTI prevalence rate among Eritrean blood donors is low, ensuring blood safety has a long way to go.

## Background

Everywhere in the world, transfusion of human blood is an essential therapeutic procedure, as there is no genuine substitution. Even though it can save human lives, in some instances it can transmit infectious diseases, which are fatal, for instance 5–10% of HIV transmission in Africa is as a result of contaminated blood transfusions [1]. Especially with the rapid increase in number of people with transfusion transmissible infections (TTI's) including HIV/AIDS, Hepatitis B and C, Human T cell Lymphotropic Virus (HTLV) I and II, Syphilis, Cytomegalovirus, Epstein Barr Virus, Brucellosis, Toxoplasmosis etc. The field of transfusion medicine has encountered a huge problem in providing safe blood and blood products; therefore there is a need to improve testing for transfusion transmissible diseases and the selection of blood donors.

In light of this, since the early 1960's blood banks as well as plasma manufacturing industries have aggressively pursued strategies to reduce the risks of the transfusion transmitted infections in both voluntary blood donors and replacement donors.

Basically we can classify the TTI's as transfusion transmitted viral, bacterial and parasitic infections [2]. The most commonly encountered transfusion infection is of viral origin. Over the last two decades, much attention has been given to the prevention of transfusion-transmitted viral infections such as HIV-1 and 2, human T cell Lymphotropic Virus (HTLV) I and II, Hepatitis C virus (HCV), Hepatitis B virus (HBV) and West Nile Virus (WNV). There is potential transmission of viruses during the “immunological window period” (i.e. the period of early infectivity when an immunologic test is non-reactive). These window periods are slightly shorter in case of the HIV virus which is the causative agent of AIDS.

HIV is an RNA retro virus belonging to the family of Lenti viruses that weakens the immune system and is the primary cause of AIDS. There are three types of HIV-1, 2 and 0. Hepatitis B virus (HBV) is a 42 nm double stranded DNA spherical particle with a double shell and Hepatitis C virus (HCV) is classified among the flaviviridae which is an RNA-single stranded virus. Both HBV and HCV cause viral hepatitis in humans. There are other two viruses that are grouped with the Hepatitis virus but they are generally associated with fecal-oral transmission, Hepatitis A virus (HAV) and E virus (HEV), have been shown to be at least occasionally transmissible via blood transfusion. Both pathogens are non-enveloped RNA viruses with a low prevalence in developed countries that have advanced environmental hygiene; a vaccine is available for HAV. The mode of transmission for HIV, HBV and HCV is the same and includes unsafe sexual contact, using sharp materials contaminated with body fluid, mother to child, and transfusion of blood and blood products. Laboratory diagnosis is based on serological tests to detect the specific antibody produced against the virus or directly detecting the antigen in body fluids and includes Enzyme Linked Immunosorbent Assay (ELISA), Radio Immunoassay (RIA), indirect immunofluorescence, immunodiffusion tests. Aside from HIV, HCV, and HBV, a number of other viral infections transmitted by transfusion of blood products have been described, even though not all have been associated with clinical manifestation. Human T cell Lymphotropic Viruses I and II (HTLV-I/II) are associated with adult T cell leukemia and HTLV-associated myelopathy/tropical spastic paraparesis. Both retroviruses have also been attributed a role in the increased risk for developing severe asthma, respiratory and urinary tract infections, uveitis and dermatitis.

Bacterial infections have the potential to be transmitted through blood transfusion, and are considered as part of TTI's. Especially syphilis a sexually transmitted infection caused by *Treponema pallidum* which is a thin delicate, tightly wound spirochate, 6-15 micrometer long with 8-14 evenly sized coils is the most important pathogen. The mode of transmission is by unsafe sexual contact, mother to child, and blood transfusion. Laboratory diagnosis is based on serological test by using specific and non-specific test. Specific test include Treponema Pallidum Haemagglutination test (TPHA), Treponema Pallidium Immobilization test (TPI), Fluorescent Treponemal Antibody test (FTA), and Enzyme Immno Assay (EIA). Non-specific test used are Venereal Disease Research Laboratory Test (VDRL) and Rapid Plasma Reagin Test (RPR). The other bacterial infections mostly are due to contamination while collecting blood during venipuncture. Frequently occurring bacterial infections are those of the normal flora associated with the gastrointestinal tract and those causing dermatitis.

Parasites are common infectious agents worldwide, and several protozoans have been shown to be transmitted via blood transfusion. Of those parasites malaria is the most prevalent one and it can be transmitted in many of the endemic area while administering transfusion. Malaria is caused by one of the four plasmodium species i.e. *P. vivax*, *P. falciparum*, *P. ovale* and *P. malariae*, which are mosquito-borne parasites that infect liver and red blood cells (RBC) causing periodic episodes of fever and flu-like symptoms, along with massive lyses of erythrocytes. These hitherto mentioned infections are generally the frequently tested infections in the developed countries. Performing these diagnostic tests in blood donors is crucial to avoid any transmission and possible death of the transfused patients [3].

TTI's can be eliminated or substantially reduced by implementing an integrated strategy for blood safety; one component of those strategies is setting up a blood transfusion service [3]. The NBTS of Eritrea is established under such considerations. It is a centralized blood transfusion services providing TTI tested blood and blood products for more than 20 referral hospitals through out the country. The testing strategy of the Eritrean NBTS includes testing markers for HIV, HBV, HCV, and Syphilis. The main objective of this study was to assess: 1) The prevalence of TTI's in Eritrean blood donors, 2) to demonstrate the difference in prevalence of TTI's among voluntary blood donors and family replacement donors.

## Methods

Retrospective review of reports compiled by the National Blood Transfusion service of Eritrea (NBTS) was extracted from the data base of the Quality Assurance department. The time frame covers from January 2006 to November 2009. The annual total blood donation with types of blood donors classified in to family replacement and voluntary donors with consecutive test result data on transfusion transmittable infection markers were included in the data. During this period a total of 29,501 units of blood were collected and out of this 23,385 (79%) units were collected from voluntary blood donors and 6116 (21%) were collected from family replacement donors. Data collection and analysis was achieved by compiling the data on excel Microsoft 2003 computing program. Simple statistical application was used to describe the result.

## Results

A total of 29,501 units of blood was collected from January 2006 to November 2009 and the proportion of blood collected from voluntary blood donors was 23,385 (79%) and the rest 6,116 (21%) were from family replacement donation (Table 1).

**Table 1:** Proportion of voluntary and family replacement donors by year (Jan 2006- Nov 2009)

Year	2006		2007		2008		Jan – Nov 2009	
Voluntary donors	4,259	71.2%	6,238	81.2%	6,449	81.9%	6,439	80%
Family Replacement	1,723	28.8%	1,443	18.8%	1,421	18.1%	1,529	20%
**Total**	**5,982**		**7,681**		**7,870**		**7,968**	

The total number of blood donors positive for serological markers of TTI was 1128 (3.8%). Of these, the number of voluntary blood donor positive for TTI markers was 817 (3.5%) and 311 (5.1%) donors were positive for TTI markers in family replacement blood donors (Table 2).

**Table 2:** Proportion of Transfusion transmitted infections (TTI) markers among voluntary and family replacement donors

TTI Markers	Voluntary Donors		Family Replacement Donors		Total Donors	
HIV	26	0.11%	26	0.43%	52	0.18%
HBV	578	2.47%	183	2.99%	761	2.58%
HCV	137	0.59%	33	0.54%	170	0.57%
Syphilis	76	0.32%	69	1.13%	145	0.49%

## Discussion

Blood transfusion is entering a new era due to the challenges posed by the increasing diversity of TTI's. This challenge is complicated due to the inescapable implication on complexity and cost particularly in resource poor settings in sub-Saharan Africa. Mathematical projection on the overall median risk of becoming infected with HIV, HBV, and HCV from a blood transfusion in sub-Saharan Africa is 1, 4.3 and 2.5 infections per 1000 units, respectively. According to this projection: if the annual transfusion requirement projected by the WHO were met, transfusion alone would be responsible for 28, 595 HBV infections, 16625 HCV infections, and 6650 HIV infections every year [4]. Currently, prevention of TTIs depends up on proper pre-donation selection of donors and serological testing of infectious markers in those donors.

The main objective of our study was to determine the sero-prevalence of TTI's including HIV, HBV, HCV and Syphilis in Eritrean blood donors. The total numbers of donors included in this study were around 29,501 and the total prevalence rates of TTI's among those donors were 3.8%. This is low when compared to the 6.9% EL-Gilany and EL-Fedawy[5] found in 1,257 voluntary donors in Egypt. The total prevalence of TTI's in voluntary donors was 3.5% and 5.1% in family replacement donors.

The prevalence of HIV in this study was 0.18% which is very low compared to many African countries 2-20% in Kenyan donors [6] and 5.9% in Ethiopian donors [7]. This is also substantiated by the overall rate in the general population was projected to be 1.3% in 2007 by UNAIDS and a subsequent study by NATCoD [8] in 2007 on ANC attendee which is considered to reflect the HIV prevalence rate in the general population gave the same rate, 1.33%.The HIV prevalence among voluntary blood donors was 0.11% and that of family replacement donors was 0.43%. Even if the HIV rate is low this study, much lower rates were reported in Egypt [5] and Saudi Arabia [9]. The decrease in the prevalence rate of HIV both on voluntary and replacement donors over the past years (Table 3) is consistent with the decrease in the prevalence rate of HIV seen in ANC attendee. If we take the study conducted in 2003 the HIV prevalence was 2.4%, in 2005 2.38% [10] and lastly in 2007, 1.33% [8, 10). This result clearly demonstrates that collecting blood from voluntary blood donors reduces the rate of HIV infection.

**Table 3:** Percentage of Transfusion transmitted infections (TTI) markers for voluntary and family replacement donors by year

Year	TTI MARKERS							
	
	HIV		HBV		HCV		Syphilis	
	Vol	F Rep	Vol	F Rep	Vol	F Rep	Vol	F Rep
	
2006	0.14%	0.56%	2.53%	3.25%	0.14%	0.2%	0.6%	1.28%
2007	0.14%	0.55%	2.53%	2.84%	1.14%	1.4%	0.05%	0.5%
2008	0.11%	0.35%	2.48%	2.53%	0.60%	0.49	0.20%	1.12%
2009 (Jan-Nov)	0.06%	0.2%	2.34%	3.3%	0.33%	0.2%	0.53%	1.57%

Vol: voluntary; F Rep: family replacement

Hepatitis B is one of the most infectious disease, it has infected around 2 billion people world wide, including an estimated 400 million chronically infected cases [11]. It is also hyper endemic in sub-Saharan Africa and Asia [12]. In our study the prevalence rate of HBs Ag was 2.58%. This figure is higher than 1.1% found by Ejele et al. [13] in Niger delta region of Nigeria, and 2.2% found by Bhatii et al. [14] in Pakistani donors. However, other studies have shown an increased HBV rate for instance 4% in Kenya donors [15], 8.8% in Tanzania donors [16] and 4.3% in Egyptian donors [17]. The prevalence rate among voluntary blood donors was 2.47% and 2.99% in family replacement donors. Even if there is a slight difference in the rate of HBV among the blood donor categories, the over all HBV rate is very high when compared to the prevalence of HIV, HCV and Syphilis among the same group of donors. In our opinion the reason behind the high rate of HBV is most probably the high infectivity potential of the virus, immunization status, cultural practices which could expose to HBV infection like circumcisions, tattooing, blood letting exercises to treat different diseases.

From this study, the prevalence rate of HCV was 0.57%, this is low when compared to 2.7% in Egypt [5], 0.5-0.97% in Iran [17] and 1.5% in Tanzania [16]. But it is a bit higher when compared to 0.2% in Kenya [15] and Elfaki et al., [18] found no cases of HCV infection in the 260 Sudanese blood donors they studied. Curiously, the prevalence rate of HCV in voluntary blood donors was 0.59% which is a bit higher than the rate in family replacement donors which was 0.54%. The low prevalence of HCV when compared to HBV might be due to the fact that HCV is less infective when compared to HBV and HCV is transmitted primarily through transfusion of blood or blood products, intravenous drug abuse and needle sharing which are not common in Eritrea.

The seroprevalence of syphilis was 0.49%. This figure is higher when compared to 0.2% among blood donors in Niger delta of Nigeria [19] and 0% among Iranian donors [20]. Nevertheless, it is low compared to 7.5% found by Adjei et al. [21] among Ghanaian blood donors and 12.7% found by Matee et al [16] among Tanzanian donors. The prevalence of syphilis in the general population is not studied but there have been three studies to assess the prevalence of Syphilis in ANC attendee which more or less are considered to represent the prevalence rate of syphilis in the general population. In 2003 the prevalence of Syphilis was estimated to be 1.6%, in 2005 it was 2.4% [10] and 1.1% in 2007 [8]. The wide difference in Syphilis infection rate and the inconsistent prevalence rate seen at the national level might be due to the use of screening tests alone which might give a falsely high positive result. The rate among voluntary donors was 0.32%, which is low when compared to the 1.13% in family replacement donors.

## Conclusion

The reduced TTI prevalence rate among Eritrean donors is an encouraging sign, which shows the effectiveness of the changes introduced in the NBTS in line with the WHO strategy for blood safety. This is also enhanced by the over all decline in the rate of HIV among the general population and also an increase in the proportion of voluntary blood donors, which was 80% in 2009. Even if we don't have the data on the proportion of repeating voluntary blood donors, we think that it will be very important for the NBTS to focus on such blood donors to further enhance the safety of blood. The HBV rate on both voluntary and replacement donors is very high compared to HIV, HCV, and Syphilis and this needs further investigation including studying the prevalence rate of HBV in the general population to address the issue. Finally even though the prevalence of HIV, HBV, HCV and Syphilis is low, ensuring blood safety has long way to go.
